# A PVDF/g−C_3_N_4_-Based Composite Polymer Electrolytes for Sodium-Ion Battery

**DOI:** 10.3390/polym15092006

**Published:** 2023-04-24

**Authors:** Kewei Shu, Jiazhen Zhou, Xiaojing Wu, Xuan Liu, Liyu Sun, Yu Wang, Siyu Tian, Huizhu Niu, Yihao Duan, Guangyu Hu, Haihua Wang

**Affiliations:** 1 Xi’an Key Laboratory of Advanced Performance Materials and Polymers, Shaanxi Key Laboratory of Chemical Additives for Industry, Shaanxi University of Science and Technology, Xuefu Road, Weiyang District, Xi’an 710021, China; 2 Department of Materials Science and Engineering, Korea University, Seoul 02841, Republic of Korea; 3 College of Chemical Engineering, Shaanxi Institute of Technology, Xi’an 710300, China

**Keywords:** graphitic C_3_N_4_, composite solid polymer electrolyte, ionic conductivity, sodium metal battery, electrochemical performance

## Abstract

As one of the most promising candidates for all-solid-state sodium-ion batteries and sodium-metal batteries, polyvinylidene difluoride (PVDF) and amorphous hexafluoropropylene (HFP) copolymerized polymer solid electrolytes still suffer from a relatively low room temperature ionic conductivity. To modify the properties of PVDF-HEP copolymer electrolytes, we introduce the graphitic C_3_N_4_ (g−C_3_N_4_) nanosheets as a novel nanofiller to form g−C_3_N_4_ composite solid polymer electrolytes (CSPEs). The analysis shows that the g−C_3_N_4_ filler can not only modify the structure in g−C_3_N_4_CSPEs by reducing the crystallinity, compared to the PVDF−HFP solid polymer electrolytes (SPEs), but also promote a further dissociation with the sodium salt through interaction between the surface atoms of the g−C_3_N_4_ and the sodium salt. As a result, enhanced electrical properties such as ionic conductivity, Na^+^ transference number, mechanical properties and thermal stability of the composite electrolyte can be observed. In particular, a low Na deposition/dissolution overpotential of about 100 mV at a current density of 1 mA cm^−2^ was found after 160 cycles with the incorporation of g−C_3_N_4_. By applying the g−C_3_N_4_ CSPEs in the sodium-metal battery with Na_3_V_2_(PO_4_)_3_ cathode, the coin cell battery exhibits a lower polarization voltage at 90 mV, and a stable reversible capacity of 93 mAh g^−1^ after 200 cycles at 1 C.

## 1. Introduction

The low reserve, uneven distribution and rising costs of lithium resources are becoming vexing problems for the currently dominant lithium-ion batteries (LIBs) [1]. Therefore, as a promising substitution for LIBs, sodium-ion batteries (SIBs) are attracting more and more attention because they have almost the same electrochemical working principles as LIBs and the abundant reserve and wide distribution of sodium element in the earth’s crust [2,3]. In the existing commercial sodium-ion batteries, the highly volatile and flammable organic electrolyte still has safety issues, as to cause a fire or even an explosion, and those disadvantages become more and more serious as the energy density increases. Additionally, for a purpose of controlling costs and improving the performance of SIBs, a common method is to replace carbon with a sodium-metal anode to form sodium-metal batteries (SMBs). However, even though the cost is controlled and performance is enhanced in SMBs, it arouses an even higher safety concern regarding the formation of sodium dendrites [4]. Therefore, all-solid-state batteries is developed using solid-state electrolytes and are considered to be the next-generation batteries due to an excellent balance between high performance and safety [5,6]. Among the various types of solid electrolytes, polymer solid electrolytes play a critical role, since they have merits of light weight, non-flammability and easy processability. More importantly, the good flexibility can alleviate the volume change of the battery during cycling and prevent dendrites growth, and also enables the feasibility of a portable battery system [7,8].

PVDF and its copolymers are the ideal matrix for polymer electrolytes due to many advantages, including corrosion/temperature resistance, easy film formation, high mechanical strength, and high dielectric constant [9,10]. However, as a semicrystalline polymer, the chain segment motion in PVDF is restricted, resulting in relatively low room temperature (RT) ionic conductivity even after copolymerization with amorphous HFP. [11,12] To increase the conductivity of PVDF−HFP copolymerized electrolytes in RT, considerable efforts have been devoted using various approaches, including incorporating nanofiller [13], adding plasticizer [14] and compositing with ceramic solid electrolytes [15]. Among them, the incorporation of nanofiller to prepare composite solid-state electrolytes (CSPEs) has proved its value by a combination of good interfacial stability, improved RT ionic conductivity and lower safety risks.

Graphitic C_3_N_4_ (g−C_3_N_4_) has a 2D stacked structure with residual -NH_2_ or -NH on the surface. Owing to its negligible electronic conductivity, rich Lewis basic N atoms, and intrinsic polarity [16], g−C_3_N_4_ can be a promising candidate as a nanofiller in polymer electrolytes. The rich N atoms on the surface of g−C_3_N_4_ can interact with the sodium salts in the polymer and increase the ionization degree of the sodium salts. Furthermore, the defects on the surface of g−C_3_N_4_ can serve as potential channels for the vertical transport of metal ions, further enhancing the ion transport ability [17]. A large interaction between g−C_3_N_4_ nanosheets and polymer matrix reduces the relative slip phenomenon, thereby the enhanced mechanical properties of the electrolyte presented [9]. The effect of g−C_3_N_4_ nanofiller on the electrical and mechanical properties of solid polymer electrolyte was firstly revealed in 2019 by Sun et al. [17]. The prepared PEO/g−C_3_N_4_ Li-ion solid polymer electrolyte presented improved ionic conductivity of up to 10^−5^ S cm^−1^ level at 30 °C, and a maximum 65.7 MPa tensile modulus. Such composite polymer electrolyte, when coupled with LiFePO_4_ positive electrode, can deliver high initial capacity of 161 mAh g^−1^ at 0.2 C under 60 °C and remained at 155 mAh g^−1^ after 100 cycles. Yang et al. further developed the PEO/g−C_3_N_4_ Li-ion CSPE by regulating the microstructure via thermal annealing [18]. The decrease crystallinity rendered the CSPE with RT ionic conductivity of 1.76 × 10^−5^ S cm^−1^, and capacity retention ratio of 81% after 200 cycles at 1 C at 80 °C when used in LiFePO_4_/Li battery. The structure of g−C_3_N_4_ can also be engineered, to regulates the Li^+^ distribution in the CSPE and mitigating the Li dendrite formation. As a result, the PEO/porous g−C_3_N_4_ CSPE showed 10^−4^ S cm^−1^ level ionic conductivity and low capacity decay of 0.05% per cycles in LiFePO_4_/Li battery [19].

In this work, we propose for the first time the preparation of composite CSPEs for SIBs by using g−C_3_N_4_ nanosheets as a nanofiller in PVDF−HFP/NaClO_4_ SPEs. Owing to the influence of g−C_3_N_4_ on the sodium salt dissociation and the crystallization of the polymer matrix [20], the mechanical properties, ionic conductivity and thermal stability of the CSPE were successfully improved. In particular, by applying CSPEs in the sodium metal battery with Na_3_V_2_(PO_4_)_3_ (NVP) cathode, electrochemical performance, cycling stability is optimized due to accelerated Na^+^ transport and suppressed Na dendrites formation.

## 2. Materials Preparation, Battery Assembly and Characterization Methods

### 2.1. Preparation of g−C_3_N_4_ Nanosheets

The g−C_3_N_4_ was synthesized by thermal oxidation etching by a well-known procedure. In detail, dicyandiamide (C_2_H_4_N_4_, AR grade, Aladdin) was heated at 550 °C for 3 h in the air with a ramp rate of 5 °C min^−1^. As result, a pale-yellow powder was obtained and ground for further usage [12,21].

### 2.2. Preparation of g−C_3_N_4_ CSPE and Assembly of Coin Cell Battery

The solid polymer electrolytes and composite solid polymer electrolytes were prepared by the solution casting method. Specifically, PVDF−HFP (M_W_ = 6 × 10^5^, Aladdin), NaClO_4_ (≥ 98.0%, anhydrous, Aladdin) and g−C_3_N_4_ were dried under vacuum, stored in an argon-filled glove box (H_2_O and O_2_ content below 0.1 ppm, Mikrouna) before used [17]. To synthesize CSPEs, 1 g PVDF−HFP, 0.05 g g−C_3_N_4_, and 0.3 g NaClO_4_ were dissolved in 20 mL N-methyl-2-pyrrolidone (NMP, 99.9%, Aladdin). After ultrasonication treatment for 30 min, magnetic stirring was performed for 18 h to obtain a uniform mixed dispersion. Then, the dispersion was cast on a polytetrafluoroethylene (PTFE) mold and vacuum-dried at 60 °C for 48 h to obtain a film with a thickness of 0.056–0.114 mm. Initially, the PVDF−HFP/NaClO_4_ electrolyte films were transparent and colorless. After combined with g−C_3_N_4_, the color gradually shifted to pale yellow (Figure S1). This is an implication of the formation of the g−C_3_N_4_ CSPEs. For comparison, SPE without g−C_3_N_4_ and neat PVDF−HFP film was prepared by the same procedure. All obtained membranes were stored in an argon-filled glove box.

For the preparation of a Na_3_V_2_(PO_4_)_3_ (NVP) cathode, the active ingredient Na_3_V_2_(PO_4_)_3_ (99.9%, Guangdong Canrd New Energy Technology Co., Ltd.), Ketjen Black (KB, ECP600JD, Lion) and sodium alginate binder (200 ± 20 mPa s, Guangdong Canrd New Energy Technology Co., Ltd., Guangdong, China) (weight ratio 8:1:1) were ground with the addition of water to form a slurry product. Then the slurry product was coated on the carbon-coated aluminum foil (Hefei Kejing Materials Technology Co., Ltd., Hefei, China), and dried in a vacuum at 60 °C for 12 h.

The feasibility of PVDF−HFP/g−C_3_N_4_/NaClO_4_ CSPEs in solid-state sodium metal batteries was investigated by fabricating the coin cell battery assembled with Na metal anodes and a NVP cathode (Na|CSPEs|NVP) [22,23,24]. The performance of coin cells, including charge-discharge cycling performance and galvanostatic cycling performance, was tested at room temperature using the LAND battery testing system (Wuhan Landian LAND-CT3001A).

### 2.3. Characterization

The structures of g−C_3_N_4_, PVDF−HFP, PVDF−HFP/NaClO_4_ SPEs and PVDF−HFP-g−C_3_N_4_-NaClO_4_ CSPEs were characterized by X-ray powder diffractometer (XRD, Rigaku MiniFlex 600) and Fourier transform infrared spectra (FTIR, Bruker VECTOR-22). For XRD, the diffraction angle ranged from 2θ = 10°~70°, and the scan rate was 5° min^−1^. For FTIR, the samples were recorded in the range of 400~4000 cm^−1^ to analyze the interactions between groups in the polymer. The tensile samples of PVDF−HFP SPEs and g−C_3_N_4_ CSPE were stretched in a gauge length of 35 mm × 2 mm on a universal testing machine (Shandong Wanchen, UTM-4000) with a 50 N sensor to evaluate their mechanical properties with a stretching rate of 10 mm min^−1^ in the thickness of 80–100 μm. The thermal stability of PVDF−HFP SPEs and g−C_3_N_4_ CSPE samples were determined by differential scanning calorimeter (DSC, TA Q2000) and Thermogravimetric Analysis (TGA, TGA-55) in sealed aluminum crucibles and heated at a rate of 10 °C min^−1^. The surface morphology analysis of polymer electrolytes was observed with a scanning electron microscope (SEM, FEI Verios 460) and atomic force microscopy (AFM, Rigaku SPI3800N/SPA400).

### 2.4. Electrochemical Test

The ionic conductivity of solid electrolytes was determined by the electrochemical impedance spectroscopy (EIS) test on an electrochemical workstation (Ametek PARSTAT3000A-DX). The electrolyte was sandwiched between two stainless steel plates. The frequency range was between 10^−1^ and 10^6^ Hz and the temperature range was from 25 to 80 °C. The ionic conductivity of polymer electrolyte was calculated by the following equation:

σ = L/R × S

where L is the thickness of the polymer electrolyte, R is the bulk resistance of the polymer electrode which can be obtained from the EIS test, and S is the area of the electrolyte.

Linear sweep voltammetry of g−C_3_N_4_ CSPE and SPE were tested on an electrochemical workstation (Ametek PARSTAT3000A-DX) at a scan rate of 5 mV s^−1^ on Na/SPE/stainless steel equipment prepared using CR 2032 coin cell. The sodium ion transfer number of g−C_3_N_4_ CSPE was calculated from the Bruce-Vincent-Evans equation:

t(_Na+_) = I_SS_ (△V − I_0_R_0_)/I_0_ (△V − I_SS_ R_SS_)

ΔV is the polarization voltage (ΔV = 10 mV), I_0_ and R_0_ are the initial current and interfacial resistance before polarization, respectively. I_ss_ and R_ss_ are the steady-state current and interfacial resistance after polarization, respectively.

## 3. Results and Discussion

### 3.1. XRD Analysis

**Figure 1:**
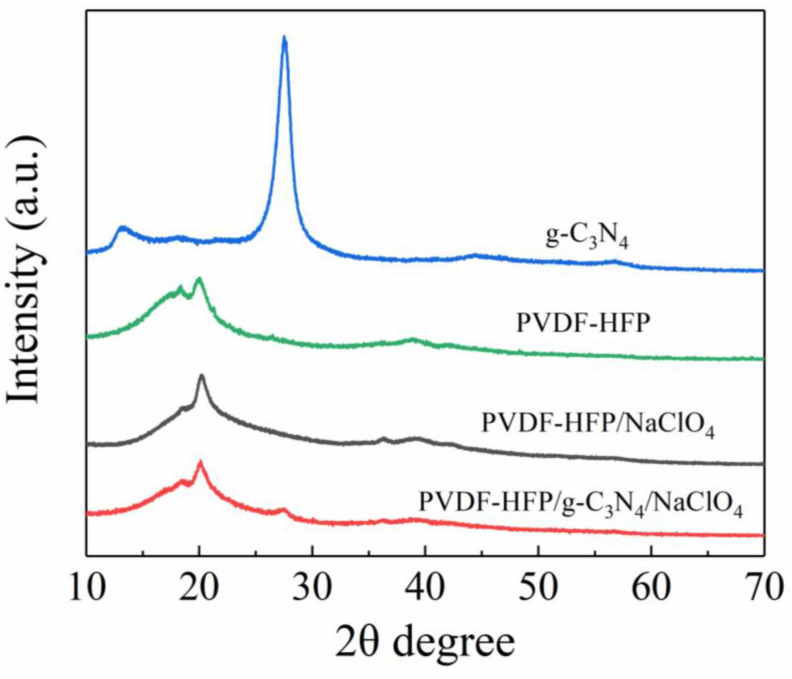
XRD patterns of g−C_3_N_4_ powder, PVDF−HFP copolymerized electrolyte, PVDF−HFP/NaClO_4_ SPEs and the PVDF−HFP/g−C_3_N_4_/NaClO_4_ CSPEs.

To characterize and investigate the composition of CSPEs, the crystallinity of g−C_3_N_4_ powder, PVDF−HFP copolymerized electrolytes, PVDF−HFP/NaClO_4_ SPEs and PVDF−HFP/g−C_3_N_4_/NaClO_4_ CSPEs was analyzed by XRD in Figure 1. The thickness of SPEs and CSPEs are prepared in the range of 70 to 100 μm (Figure S2), since it’s the common value to lower the battery impedance and ensure flexibility of the membrane [25]. Since PVDF is a semicrystalline polymer and has two main crystal phases, α and β, two partially overlapped diffraction peaks are observed in PVDF−HFP/NaClO_4_ at 18.5°and 20.3°, which are reflected from the crystal plane of α (100) and β (110) (200) phase, respectively [26,27]. In a comparison of the g−C_3_N_4_ powder and PVDF−HFP/g−C_3_N_4_/NaClO_4_ CSPEs, a tiny g−C_3_N_4_ characteristic peak at 27.6° appeared in CEPEs and did not have a significant change before and after hybridization (Figure 1 inset), indicating that the presence of g−C_3_N_4_ and good compatibility in the preparation process [12,20]. Furthermore, by calculation, the crystallite size is reduced from 37 nm in PVDF−HFP/NaClO_4_ SPEs to 23 nm in PVDF−HFP/g−C_3_N_4_/NaClO_4_ CSPEs according to the Williamson-Hall Methods [28,29]. The Segel crystallinity index (CI) for the polymer electrolyte was calculated from the ratio of the total intensity of the α (100) and β (110) (200) phase and the intensity of the amorphous α phase peak, which is CI= (I_total_ − I_amorphous_)/I_total_ [30,31]. The CI for PVDF−HFP/NaClO_4_ SPEs and PVDF−HFP/g−C_3_N_4_/NaClO_4_ CSPEs is 0.62 and 0.55. Therefore, it is obvious that the addition of g−C_3_N_4_ in PVDF−HFP/NaClO_4_ decreased the crystallization, as revealed by a weakened amorphous peak intensity. The high degree of crystallinity of PVDF−HFP results in low ionic conductivity of its corresponding solid electrolyte at room temperature. Meanwhile, the broader PVDF−HFP signal in CSPEs (inset) indicates lowered degree of crystallinity, which would lead to higher RT ionic conductivity. The XRD confirmed that the addition of g−C_3_N_4_ nanofiller can reduce the crystallinity of PVDF−HFP and increase the amorphous region, thereby enhancing the mobility of sodium ions [18].

### 3.2. FTIR Spectra Analysis

**Figure 2:**
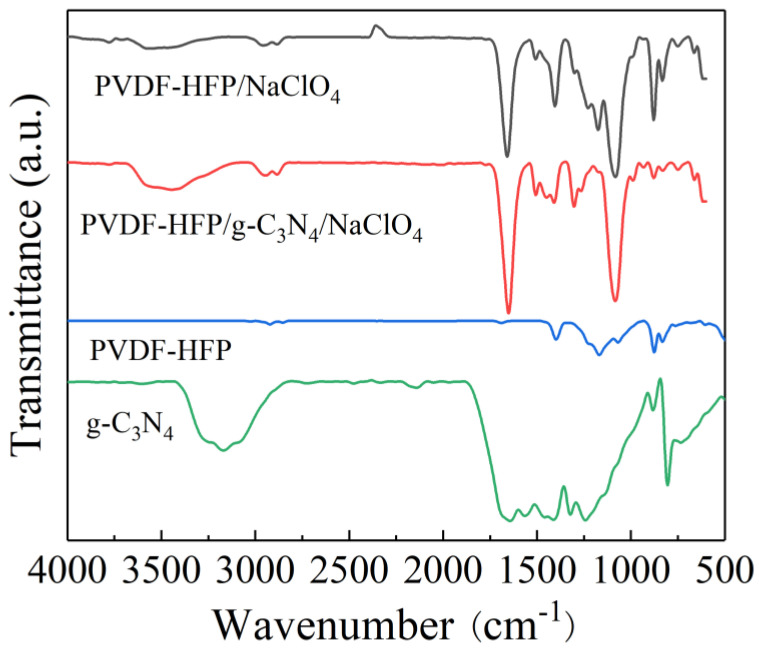
FT−IR spectra of g−C_3_N_4_, PVDF−HFP copolymerized electrolyte, PVDF−HFP/NaClO_4_ SPEs and PVDF−HFP/g−C_3_N_4_/NaClO_4_ CSPEs.

In Figure 2, the FTIR spectra of PVDF−HFP/g−C_3_N_4_/NaClO_4_ CSPEs in the range of 500–4000 cm^−1^ are shown. After adding nanofillers, the broad peak emerges at 3450 cm^−1^ in PVDF−HFP/g−C_3_N_4_/NaClO_4_ CSPEs, which is associated with the residual N-H bonded structure on the surface of g−C_3_N_4_ [32]. Characteristics peaks of the C-N(-C)-C and bridging C-NH-C bonded structures appear between 1800–900cm^−1^ [33]. The peak at 744–875 cm^−1^ and 838 cm^−1^ can be considered as the α and amorphous phase of crystalline PVDF−HFP, respectively [34]. The two characteristic peaks weakened or even disappeared in PVDF−HFP/g−C_3_N_4_/NaClO_4_ CSPEs, indicating the interaction between g−C_3_N_4_ nanosheets and polymer matrix [35]. The rich N atoms on the surface of g−C_3_N_4_ can be considered a Lewis base [18]. Thus, the interaction between N-Na leads to an increase in the dissociation of the sodium salt, which has the ability to enhance ionic conductivity.

### 3.3. Stress-Strain Tests

**Figure 3:**
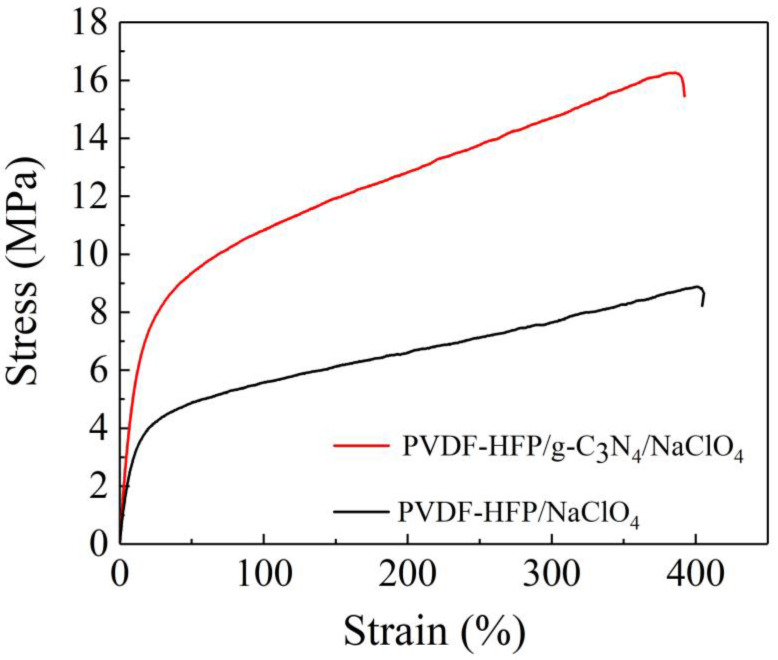
Stress-strain curves PVDF−HFP/NaClO_4_ and PVDF−HFP/g−C_3_N_4_/NaClO_4_ CSPEs.

Mechanical properties of PVDF−HFP/NaClO_4_ SPEs and PVDF−HFP/g−C_3_N_4_/NaClO_4_ CSPEs are shown in Figure 3, which is a critical parameter to evaluate the performance of polymer solid electrolytes by inhibiting the formation of Na dendrites [36]. The tensile strength of the CSPEs and SPEs are 16.2 MPa and 8.89 MPa at the maximum of strain, separately. It can be seen that the tensile strength gradually increases by 7.38 MPa, and the corresponding Young’s modulus increased from 40.9 MPa to 66.8 MPa. Therefore, it was expected that the PVDF−HFP/g−C_3_N_4_/NaClO_4_ CSPEs prevented the sodium dendrites because of the larger tensile modulus which can delay the nucleation of sodium dendrites. The mechanic properties of our PVDF−HFP/g−C_3_N_4_/NaClO_4_ composite is also very consistent with that of composite solid electrolytes for sodium batteries reported elsewhere [37].

### 3.4. Thermal Stability Analysis

**Figure 4:**
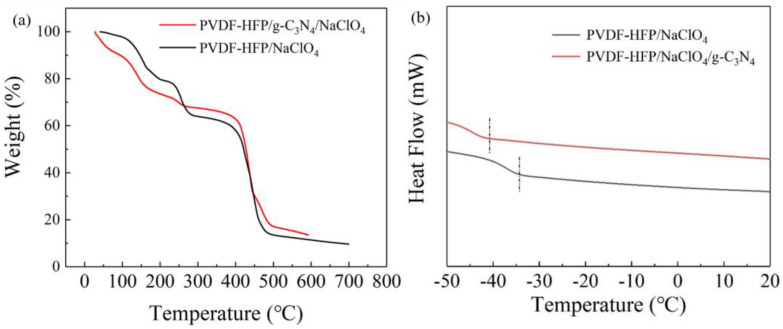
(**a**) TGA curves of PVDF−HFP/NaClO_4_ and PVDF−HFP/g−C_3_N_4_/NaClO_4_ CSPEs; (**b**) DSC curves of PVDF−HFP/NaClO_4_ and PVDF−HFP/g−C_3_N_4_/NaClO_4_ CSPEs.

Figure 4a presents the TGA curves of the polymer electrolytes. The weight loss of the electrolyte between 120–180 °C was caused by the dissociation of NaClO_4_; the rapid weight loss between 400–450 °C can be attributed to the complete decomposition of the PVDF−HFP polymer. Compared with the process of weight loss of the PVDF−HFP/g−C_3_N_4_/NaClO_4_ CSPEs and PVDF−HFP/NaClO_4_ SPEs, a slower dissociation of NaClO_4_ in the CSPEs can be found, which indicates that the CSPEs exhibited higher thermal stability. Thereby, the safety of battery with incorporated g−C_3_N_4_ can be improved. The solvent residue in the PVDF−HFP CSPEs and SPEs was approximately 11%, as revealed by comparing TGA curves of PVDF−HFP powder and PVDF−HFP membrane prepared without Na salt and nanofiller (Figure S4). In Figure 4b, the T_g_ of PVDF−HFP/NaClO_4_/g−C_3_N_4_ was −41 °C, which was decreased compared to that of PVDF−HFP/NaClO_4_ (−34 °C). As also revealed by DSC, the melting point slightly lowered from 146 °C to 143 °C after g−C_3_N_4_ introduced (Figure S5). This indicates that the introduction of g−C_3_N_4_ reduces the crystallinity of PVDF−HFP, resulting in an improvement of the segment mobility in the electrolyte [6,34].

### 3.5. Morphology Analysis

**Figure 5:**
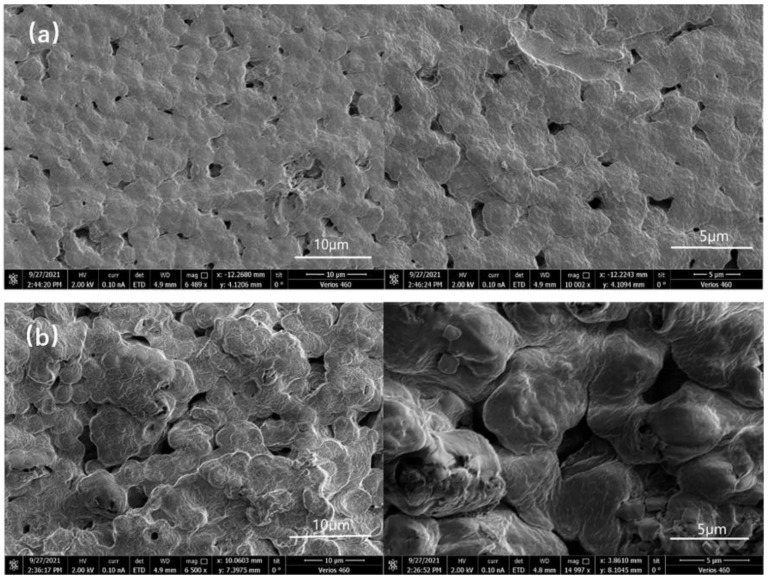

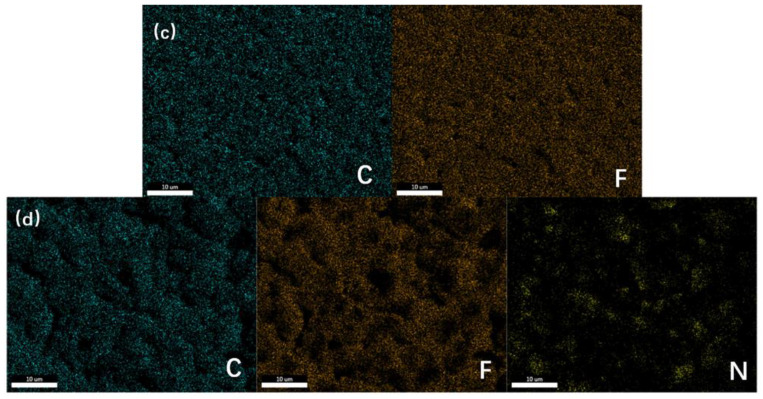
SEM images of (**a**) PVDF/NaClO_4_ and (**b**) PVDF/g−C_3_N_4_/NaClO_4_; EDS images of (**c**) PVDF/NaClO_4_ and (**d**) PVDF/g−C_3_N_4_/NaClO_4_.

The morphology of the as-prepared PVDF−HFP based CSPEs was firstly characterized by SEM. As shown in Figure 5a,b, bare PVDF SPE presents a dense and smooth surface with micrometer-sized pores. Meanwhile, the surface of CSPE with g−C_3_N_4_ has a nodular-like structure with increased micropores. The evolution in morphology suggests that the addition of g−C_3_N_4_ weakened the consistency of the polymer matrix, which greatly lowered the crystallinity of the electrolyte as previously revealed by XRD analysis. The addition of g−C_3_N_4_ was beneficial to improve the ionic conductivity of the composite solid polymer electrolyte. The corresponding EDS mapping shows that the C, F elements are uniformly distributed in SPE and CSPE film along with PVDF−HFP aggregation (Figure 5c,d). The N element signal appeared in the CSPEs, confirming the successful combination of g−C_3_N_4_ and polymer matrix.

**Figure 6:**
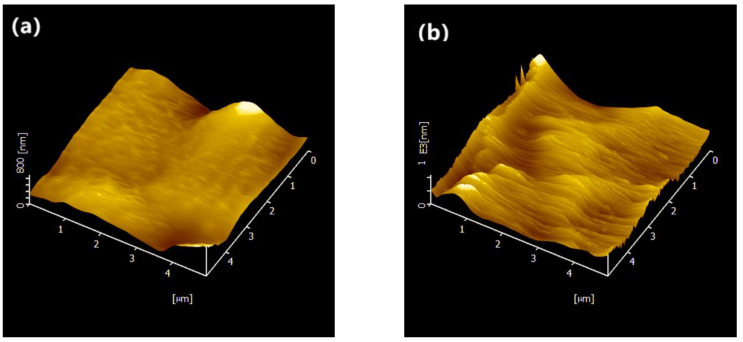
AFM images of (**a**) PVDF/NaClO_4_ and (**b**) PVDF/g−C_3_N_4_/NaClO_4_.

By further investigation, the topography images of PVDF−HFP/g−C_3_N_4_/NaClO_4_ CSPE obtained by AFM show a clear mountain-valley pattern, as shown in Figure 6. Comparing the 3D AFM images of the SPE (Figure 6a) and CSPE (Figure 6b), a clear mountain-valley pattern in the CSPE was observed, and this indicates a rough surface was formed in CSPEs. Furthermore, in 2D AFM images of the SPE and CSPE (Figure S3), the root-mean-roughness (RMS) in CSPE was calculated as 134.9 nm, which is a huge increase from 63.9 nm SPE without the nanofiller. This indicates the presence of g−C_3_N_4_ nanosheets modifies the construction in CSPEs and shows a good complexation between the polymer and the g−C_3_N_4_. No small particles are observed on the surface, indicating the g−C_3_N_4_ nanosheets have been uniformly dispersed into the host polymer.

### 3.6. The Electrochemical Characterization

**Figure 7:**
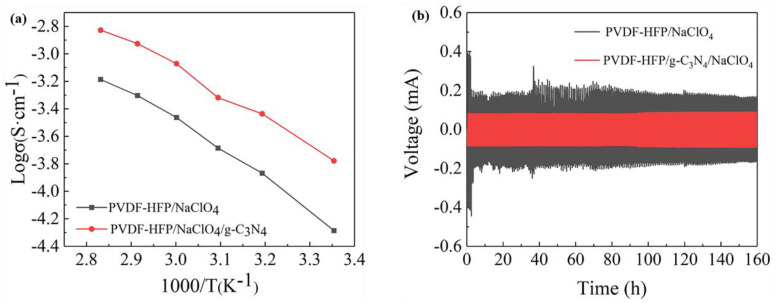
(**a**) Temperature dependence of ionic conductivities of PVDF−HFP/NaClO_4_ and PVDF−HFP/g−C_3_N_4_/NaClO_4_ CSPEs; (**b**) Galvanostatic cycling profiles of symmetric Na|PVDF−HFP/NaClO_4_|Na and Na|PVDF−HFP/g−C_3_N_4_/NaClO_4_|Na cells at a current density of 1 mA cm^−2^.

To understand the effect of g−C_3_N_4_ filler on the ionic transport capability of PVDF−HFP polymer solid electrolyte, the ionic conductivity of the CSPE was calculated based on electrochemical impedance spectroscopy (EIS) results in the frequency range of 0.01~10^6^ Hz. The Arrhenius plot of ionic conductivity in the temperature range from 25 °C to 80 °C is presented in Figure 7a. After the addition of the g−C_3_N_4_ nanofiller, the RT ionic conductivity experienced a significant improvement, from 5.17 × 10^−5^ to 1.67 × 10^−4^ S cm^−1^. The increase in ionic conductivity resulted from the enhanced segment motion of the polymer, which was owing to the decrease in crystallinity of the polymer induced by nanofiller incorporation, as demonstrated by previous characterization [13,17]. Besides, the RT ionic conductivity of our work was much higher than that of the reported PEO/g−C_3_N_4_/LiClO_4_ (1.76 × 10^−5^ S cm^−1^ at 25 °C), PEO/g−C_3_N_4_/LiTFSI solid electrolytes (1.70 × 10^−5^ S cm^−1^ at 30 °C) [17,18]. The electrochemical stability window of SPE/CSPE was determined by linear sweep voltammetry (LSV) at room temperature using SS|CSPE|Na structured coin cells. As shown in Figure S8, the electrochemical window of the PVDF−HFP/g−C_3_N_4_/NaClO_4_ composite electrolyte is 4.8 V, while the electrochemical window of the PVDF−HFP/NaClO_4_ composite electrolyte is only 3.6 V. The addition of g−C_3_N_4_ improved the stability of electrolyte due to the interaction between PVDF−HFP and the nanofiller, and thus preventing the reaction between the end groups and Na metal [18,35]. In the polymer solid electrolyte system, positive and negative ions can move at the same time, and the number of negative ion migrations is usually more significant than the number of positive ion migrations [18]. It is crucial to determine the sodium ion transport number (t_Na+_) of polymer electrolytes, since the polarization potential during recycling can effectively be reduced with a higher sodium ion mobility number [17]. Figure S6 shows the chronoamperometry and AC impedance spectra (inset) before and after polarization of the Na|SPE|Na cell. The t_Na+_ of the polymer electrolyte was then measured and calculated. CSPE has a sodium ion migration number of 0.78, while that of PVDF−HFP/NaClO_4_ composite solid electrolyte was 0.61. The interaction between nitrogen atoms and Na^+^ promotes the dissociation of sodium salts in the polymer, thus leading to higher t_Na+_. For sodium metal batteries, a higher sodium ion migration number also increases the ionic conductivity of sodium ions, thereby making the sodium metal anode more stable [12,18]. To evaluate the compatibility and the cycling stability of the interface between PVDF−HFP/g−C_3_N_4_/NaClO_4_ CSPE and sodium metal, the galvanostatic cycling performance of Na|CSPE|Na symmetric cells was performed at 1 mA cm^−2^. The behavior of Na metal deposition/dissolution can be reflected by polarization voltage during charge/discharge, as displayed in Figure 7b [21]. The cell with g−C_3_N_4_ CSPE presented low deposition/dissolution overpotential of 100 mV, and excellent cycling stability over 180 h without short circuit and polarization voltage increase. By contrast, the cell using SPE without filler has a high initial overpotential (300 mV) and exhibits instability after 40–60 h cycling due to the internal short circuit caused by sodium dendrites formation. The g−C_3_N_4_ composite promoted uniform Na deposition/dissolution, leading to improved interfacial compatibility and long-term stability and finally resulting in enhancement of battery life.

### 3.7. Battery Performance Based on PVDF−HFP/g−C_3_N_4_/NaClO_4_ CSPE

**Figure 8:**
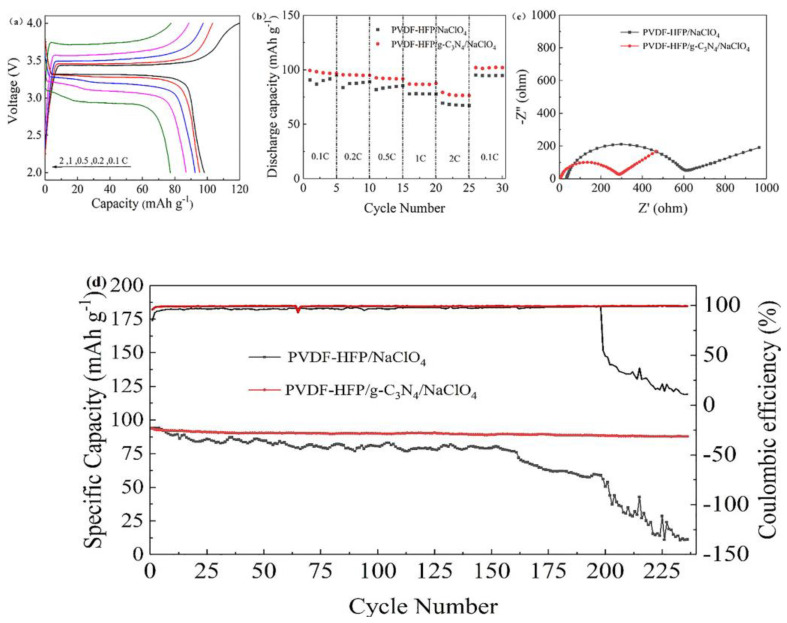
(**a**) Charge/discharge curves of Na|PVDF−HFP/g−C_3_N_4_/NaClO_4_ |NVP cell at 0.1 C(black), 0.2 C(red), 0.5 C(blue), 1 C(purple) and 2 C(green). (**b**) Rate performances of Na|PVDF−HFP/NaClO_4_|NVP and Na| PVDF−HFP/g−C_3_N_4_/NaClO_4_ |NVP cells. (**c**) EIS curves of PVDF−HFP/NaClO_4_ and PVDF−HFP/g−C_3_N_4_/NaClO_4_. (**d**) Cycling performances of Na|PVDF−HFP/NaClO_4_|NVP and Na|PVDF−HFP/g−C_3_N_4_/NaClO_4_ |NVP cells at 1C.

The performance of coin cell type battery consists of PVDF−HFP/NaClO_4_ SPE or the PVDF−HFP/g−C_3_N_4_/NaClO_4_ CSPE, Na metal anode and NVP cathode was evaluated. At the same current density of 0.1 C, the Na|PVDF−HFP/g−C_3_N_4_/NaClO_4_|NVP cell exhibits a higher specific capacity of 100.3 mAh g^−1^ than that of Na|PVDF−HFP/NaClO_4_|NVP cell (81.5 mAh g^−1^) (Figure 8b) [38,39]. Meanwhile, the Na|PVDF−HFP/g−C_3_N_4_/NaClO_4_|NVP cell exhibits a flatter charging/discharging voltage plateau and lower polarization voltage (0.09 V) than that of Na|PVDF−HFP/NaClO_4_|NVP cell (0.21 V) (Figure S7). The specific discharge capacity of Na|PVDF−HFP/g−C_3_N_4_/NaClO_4_|NVP is 100.3, 95.3, 92.5, 86.7 and 79.2 mAh g^−1^ at 0.1, 0.2, 0.5, 1 and 2 C, respectively (Figure 8a). The PVDF−HFP/g−C_3_N_4_/NaClO_4_ CSPE exhibited excellent rate capability, which was also improved compared with SPE without g−C_3_N_4_. The enhancement was ascribed to the minimized Na^+^ concentration gradient and the accelerated transport ability of Na^+^ in the PVDF−HFP/g−C_3_N_4_/NaClO_4_ CSPE. It can be seen from Figure 8c that the diameter of the semicircle with the g−C_3_N_4_ CSPE is smaller than the SPE without the g−C_3_N_4_. It indicates that the intercalation and deintercalation of sodium ions are easier.

The cycling performance at the current density of 1C is shown in Figure 8d. The cell with the g−C_3_N_4_ CSPE had an initial capacity of up to 95 mAh g^−1^, which retained at 93 mAh g^−1^ after 200 cycles. Meanwhile, the coulombic efficiency of the cells with g−C_3_N_4_ CSPE is as high as 99.5%. By contrast, the cells assembled using SPE without g−C_3_N_4_ decayed severely after 200 cycles with only 69.3% capacity retention. Therefore, the lifetime of solid-state sodium metal batteries is prolonged due to the enhanced interfacial stability between the PVDF−HFP/g−C_3_N_4_/NaClO_4_ CSE and Na metal anodes, as well as the fast Na^+^ transport.

## 4. Conclusions

In summary, a PVDF−HFP/g−C_3_N_4_/NaClO_4_ composite polymer electrolyte with excellent comprehensive properties was prepared for the first time. The process of incorporation of g−C_3_N_4_ nanofiller in PVDF−HFP to form CSPEs are characterized by measurements in the XRD and FTIR. A significant crystallinity reduction, an even distribution of g−C_3_N_4_ nanofiller in CSPEs are also found through morphological measurements in the SEM and AFM. The presence of g−C_3_N_4_ nanosheet in CSPEs provides an efficient interface with polymers, and promotes Na^+^ dissociation via the interaction of the surface N atoms with the sodium salt. Consequently, the mechanical properties and ionic conductivity are simultaneously enhanced through the thermal stability and electrochemical characterization. Regarding to the good mechanical and electrochemical properties, the PVDF−HFP/g−C_3_N_4_/NaClO_4_ CSPEs are used in the all solid-state sodium coin cell battery, Resulting a superior cycle performance and high safety performance in comparison of the coin cell battery assembled with the PVDF−HFP/NaClO_4_ SPEs. The addition of this novel filler opens up new ideas for further research on composite electrolytes, making solid-state batteries practical in large-scale energy storage, making them the development direction of next-generation rechargeable solid-state batteries.

## Data Availability

Not applicable.

## Supplementary Materials

The following supporting information can be downloaded at: https://www.mdpi.com/article/10.3390/polym15092006/s1, Figure S1: Characterization; Figure S2: Characterization; Figure S3: Characterization; Figure S4: Thermodynamic Measurement; Figure S5: Thermodynamic Measurement; Figure S6: Electrochemical Measurement; Figure S7: Electrochemical Measurement; Figure S8: Electrochemical Measurement.
