# Supplementary material for: A PVDF/g−C3N4-Based Composite Polymer Electrolytes for Sodium-Ion Battery
Source: Polymers (Basel). 2023 Apr 24;15(9):2006. doi: 10.3390/polym15092006 (PMC10181288; doi:10.3390/polym15092006)
Supplement: Supplementary file 1 [file polymers-15-02006-s001.zip › polymers-2339827-supplementary.pdf]

# Supplementary Materials

# A g-$C_3N_4$/PVDF-based composite polymer electrolytes for sodium-ion battery

## Characterization

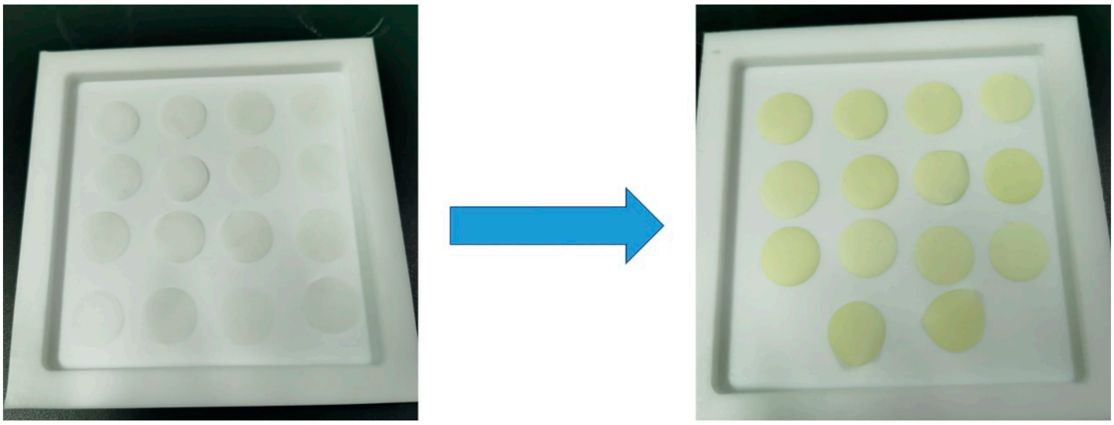

**Figure S1** Photos of color change of solid electrolyte after combination with g-$C_3N_4$

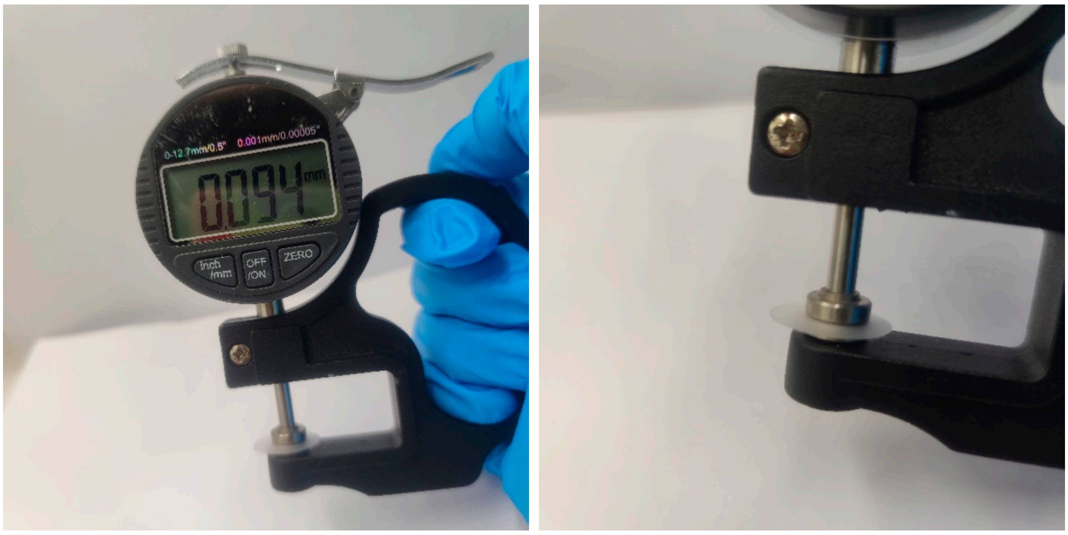


**Figure S2**. Thickness measurements for the solid electrolytes.

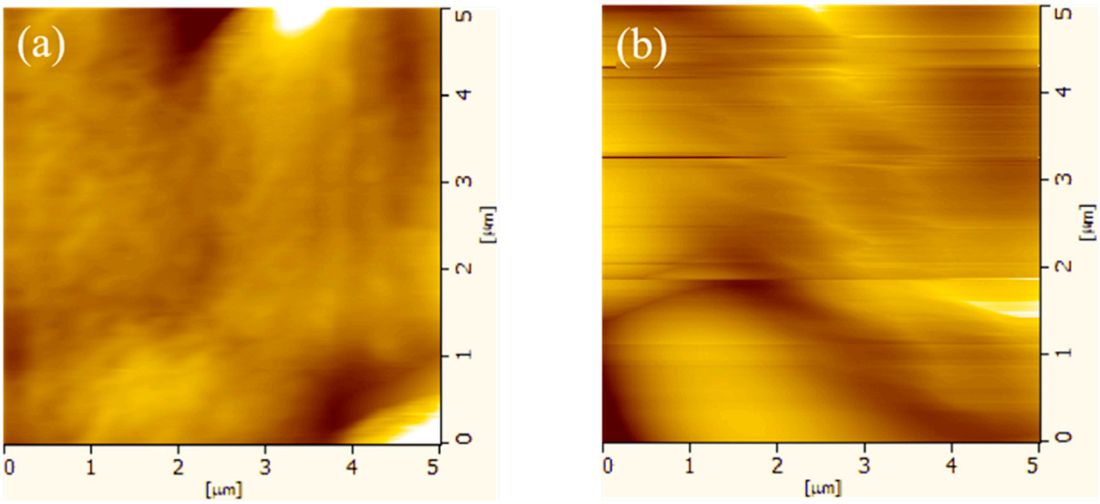


**Figure S3**. AFM height scans of (a) PVDF/$NaClO_4$ and (b) PVDF/g-$C_3N_4$/$NaClO_4$

**Thermodynamic Measurement**

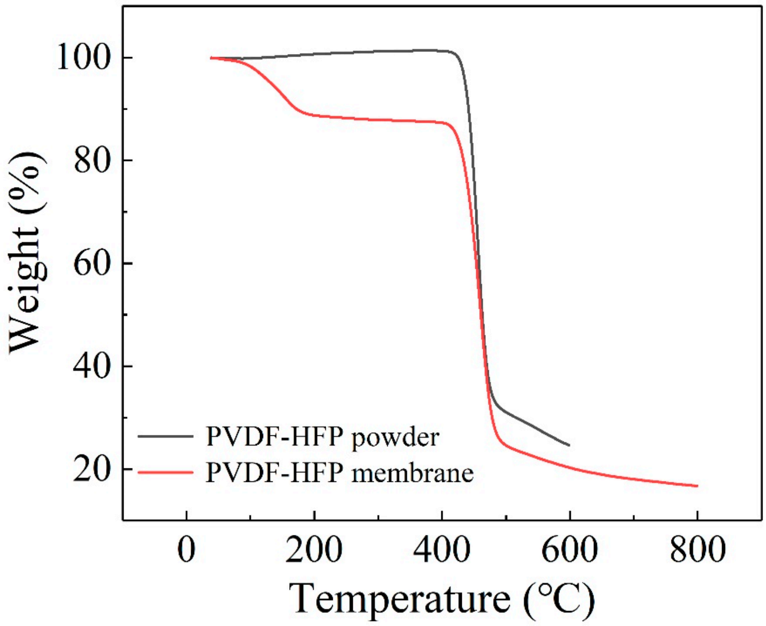


**Figure S4**. TGA curves of PVDF-HFP powder and PVDF-HFP membrane

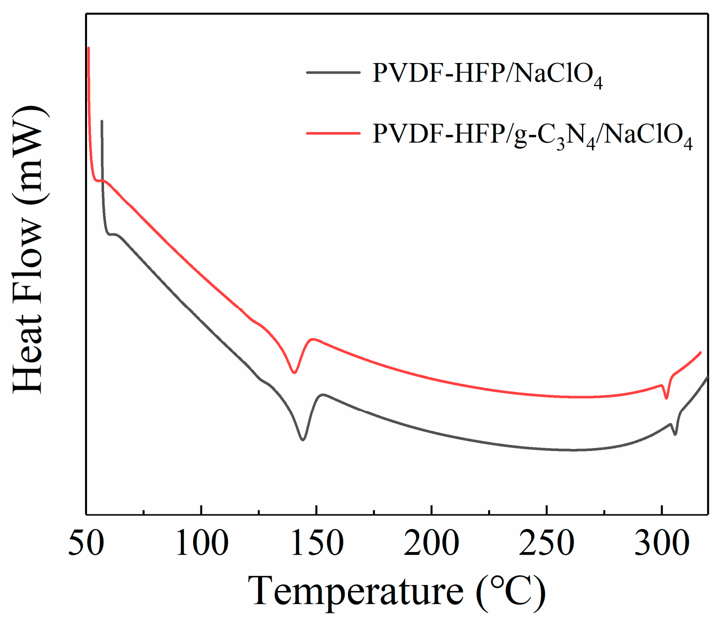


**Figure S5**. DSC curves of PVDF-HFP/$NaClO_4$ and PVDF-HFP/g-$C_3N_4$/$NaClO_4$ CSPEs

**Electrochemical Measurement**

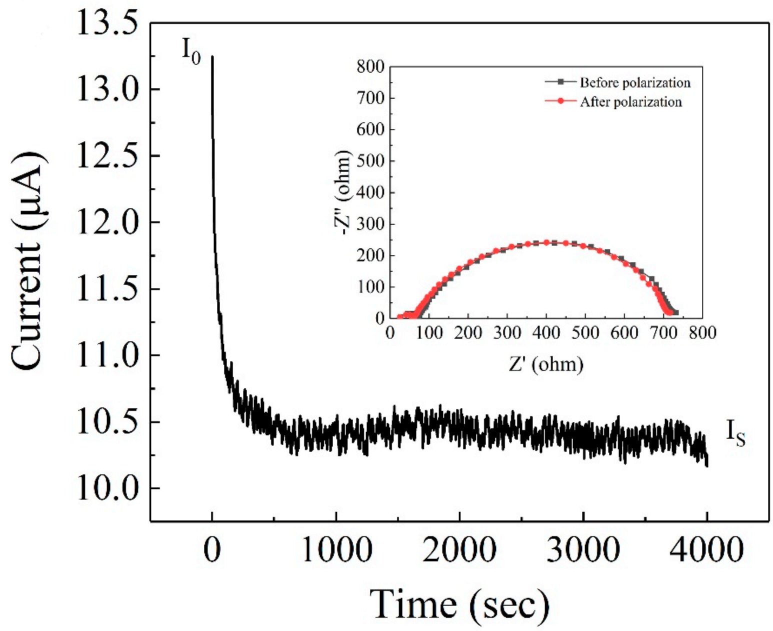


**Figure S6**. Chrono current curves and EIS before and after polarization (inset)

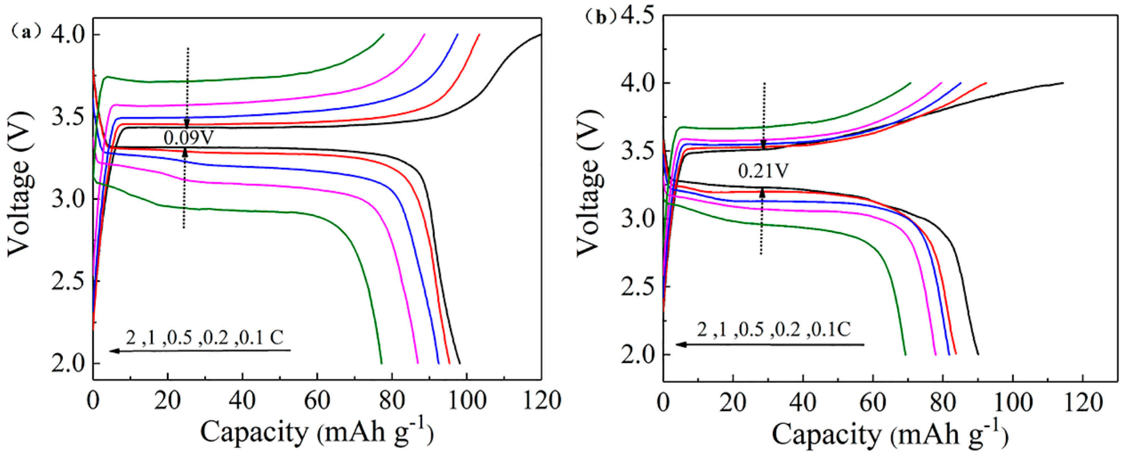


**Figure S7.** The charge and discharge curves of (a)Na| PVDF-HFP/g-$C_3N_4$/$NaClO_4$ |$Na_3V_2(PO_4)_3$ and (b)Na| PVDF-HFP/$NaClO_4$ |$Na_3V_2(PO_4)_3$ cells at 0.1 C, 0.2 C, 0.5 C, 1 C and 2 C.

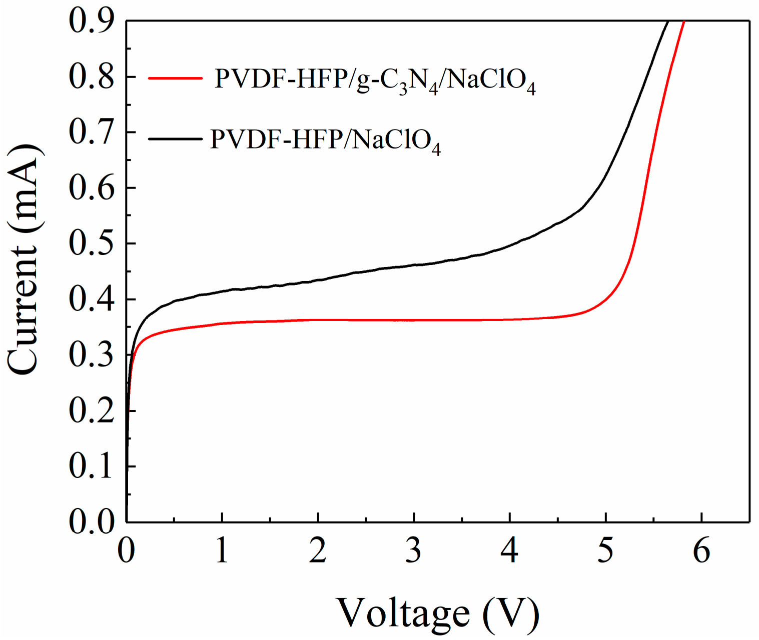


**Figure S8.** Linear sweep voltammetry curves of PVDF-HFP/$NaClO_4$ and PVDF-HFP/g-$C_3N_4$/$NaClO_4$ CSPEs
